# Supplementary material for: miR-122 removal in the liver activates imprinted microRNAs and enables more effective microRNA-mediated gene repression
Source: Nat Commun. 2018 Dec 14;9:5321. doi: 10.1038/s41467-018-07786-7 (PMC6294001; doi:10.1038/s41467-018-07786-7)
Supplement: Supplementary file 1 — Supplementary Information [file 41467_2018_7786_MOESM1_ESM.docx]

­­

Supplementary Information

miR-122 removal in the liver activates imprinted microRNAs and enables more effective microRNA-mediated gene repression

Paul N. Valdmanis, Hak Kyun Kim, Kirk Chu, Feijie Zhang, Jianpeng Xu, Elizabeth M. Munding, Jia Shen, and Mark A. Kay


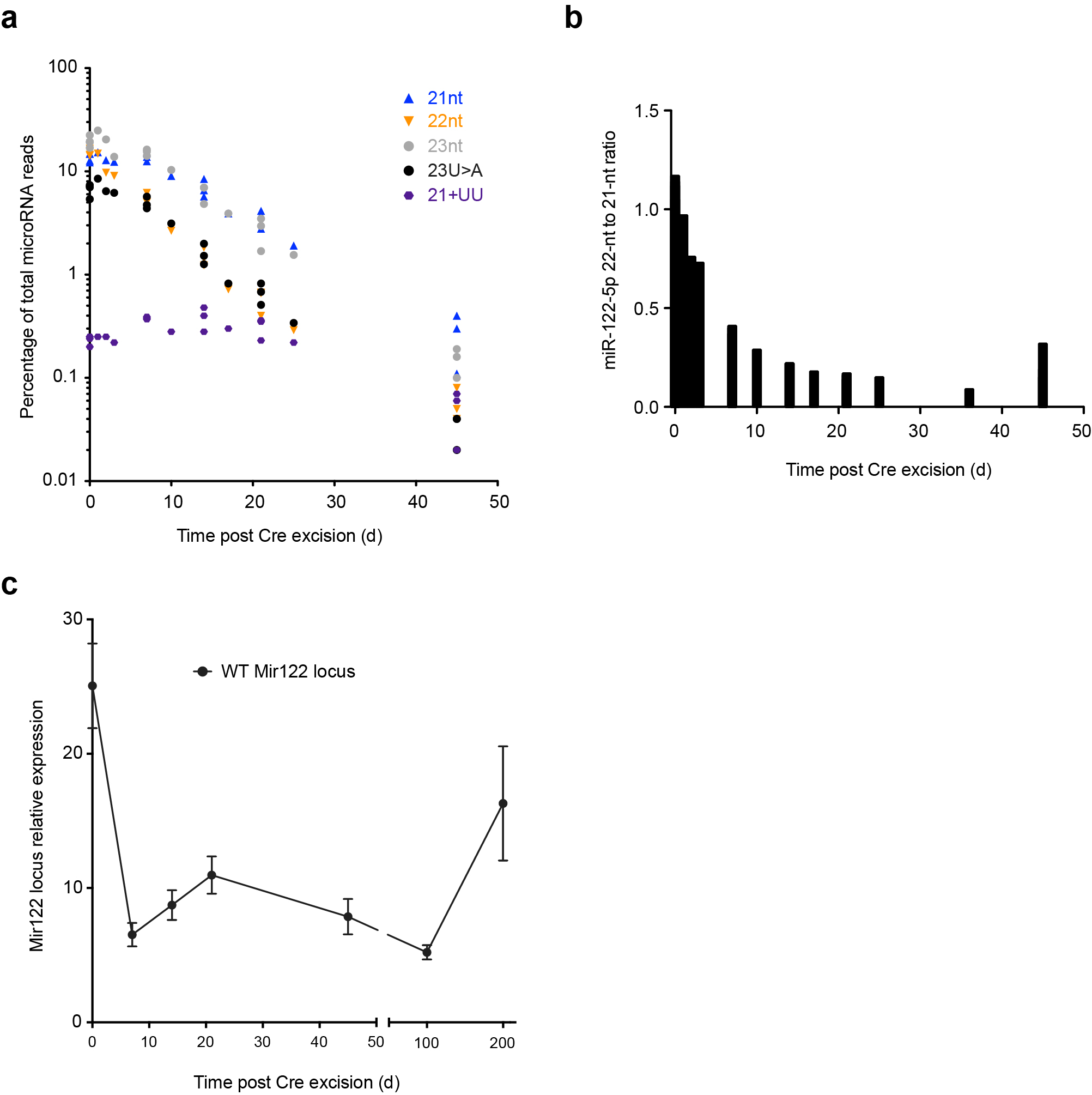


**Supplementary Figure 1** Mir-122-5p 22nt and 23U>A isoforms and 21nt and 23nt isoforms decay at similar rates. **a** Levels of common miR-122-5p isoforms at various time points after miR-122 excision relative to all microRNA species (*n* = 3 for days 0, 7, 14, 21 and 45; *n* = 1 for remaining time points). **b** 22nt to 21nt ratio of miR-122-5p at various time points after Cre-mediated miR-122 excision; replicate numbers as in (**a**). **c** Quantitative PCR of the miR-122 transcript DNA prior to locus excision by Cre.

**
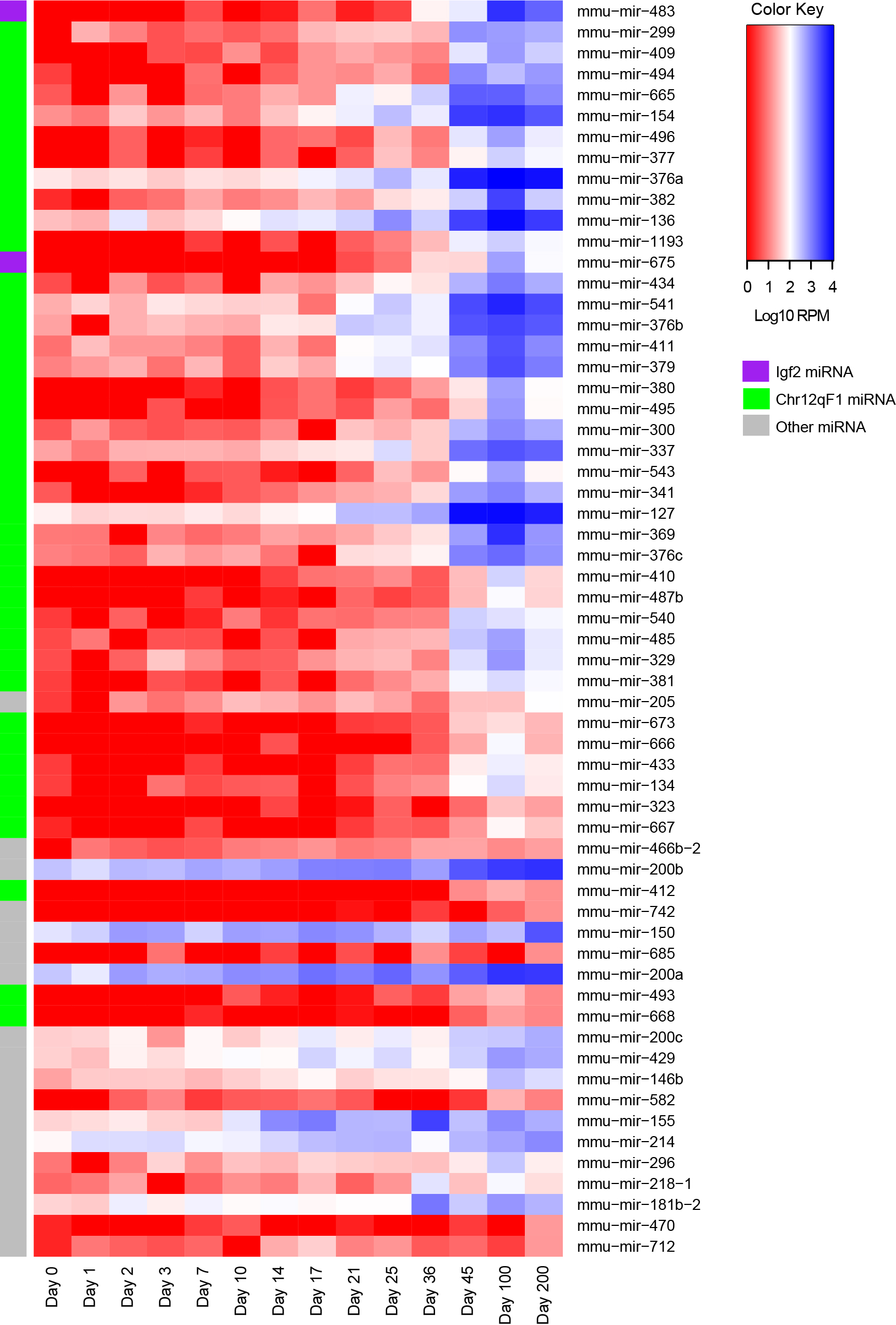
**

**Supplementary Figure 2** MicroRNAs most responsive to loss of miR-122. Mouse microRNAs are plotted in decreasing order of fold-change from 100 days post Cre mediated excision of miR-122 to day 0 (control). Log10 values of reads per million (RPM) microRNA counts are shown.


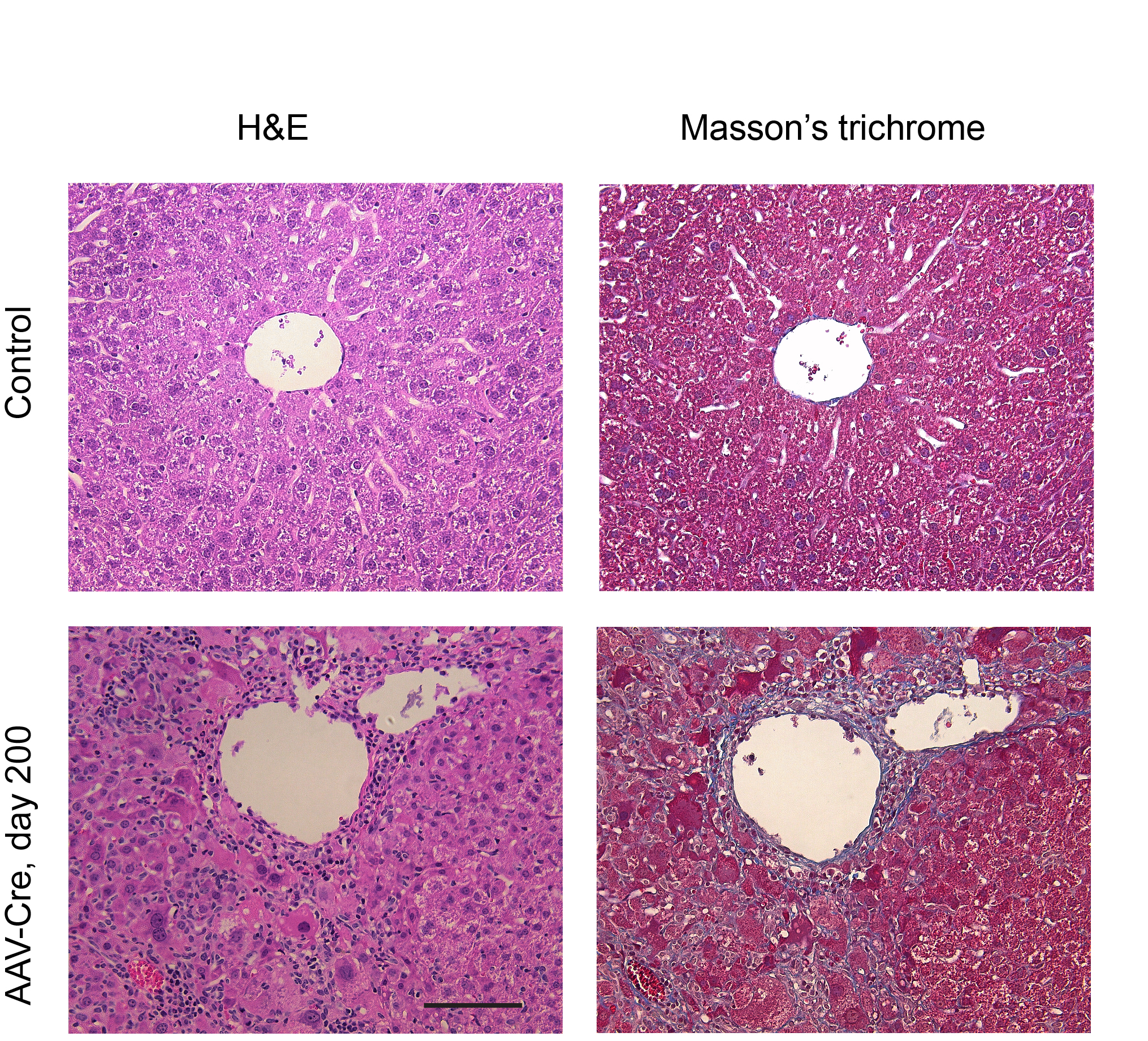


**Supplementary Figure 3** Histology of mouse liver sections 200 days after Cre mediated removal of miR-122. Masson’s trichome indicates levels of fibrosis. Scale bar: 100 μm. H&E, haematoxylin and eosin staining.

**
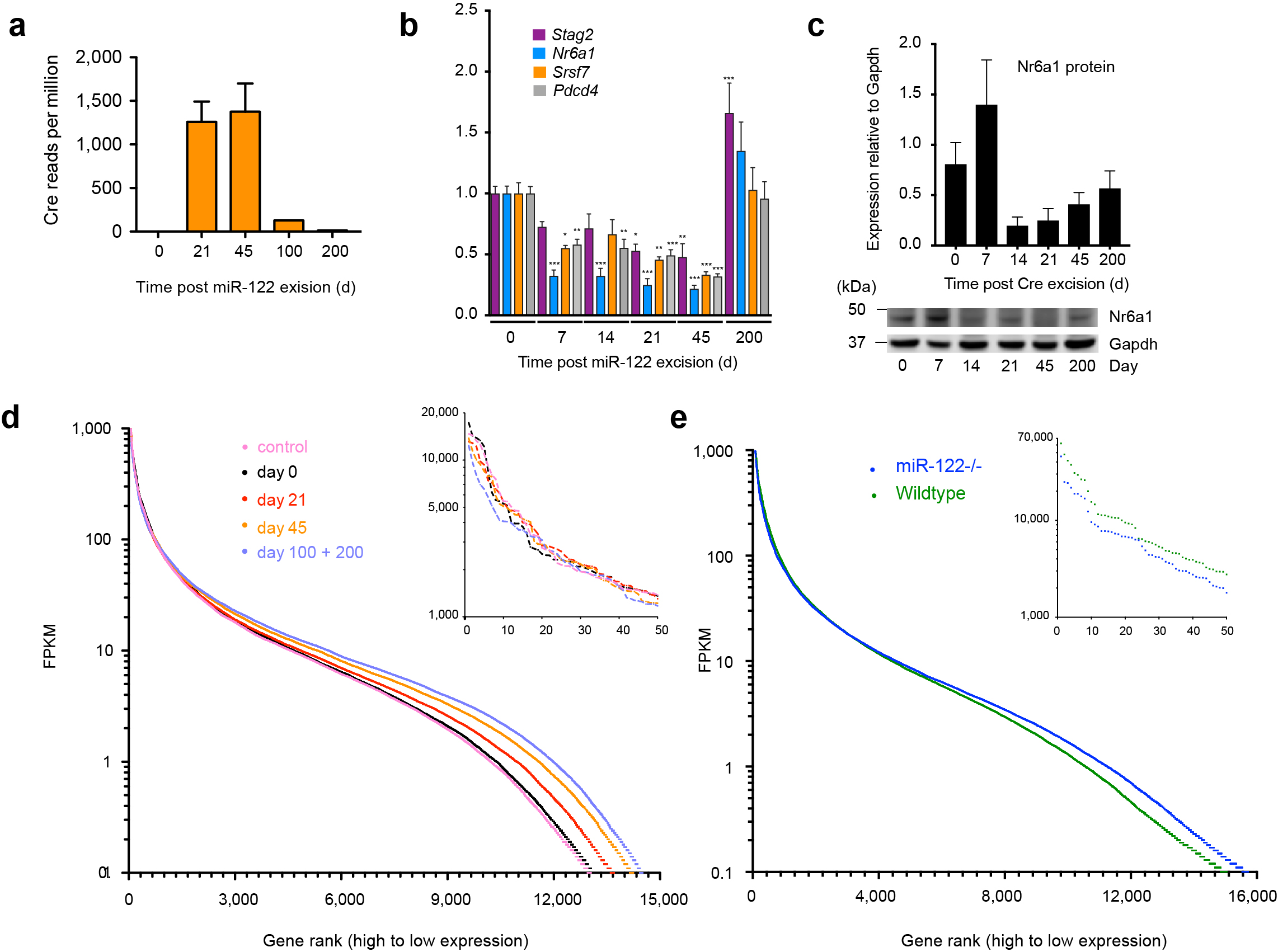
**

**Supplementary Figure 4** Rare transcripts display increased expression after miR-122 excision. **a** Reads mapping to Cre normalized per million mapped fragments to the mouse mm9 genome; *n* = 3 for 0, 21 and 45 days; *n* = 1 for 100 and 200 days; error bars are ± s.e.m. **b** qPCR of predicted target mRNAs of common microRNAs; *n* = 3 replicates per condition performed in triplicate, error bars are s.e.m.; * = p<0.05, ** = p<0.01, *** = p<0.001 by one-way ANOVA relative to day zero with Dunnett’s correction for multiple testing. **c** Western blot analysis of Nr6a1 protein levels at various points after Cre removal; *n* = 3 replicates per condition, error bars are s.e.m. **d** Sorted FPKM values are plotted based on condition. Inset, the first 50 genes are displayed. **e** Average sorted FPKM values of mRNA expression from wildtype and *Mir122* knockout mice from Luna *et al^1^*.


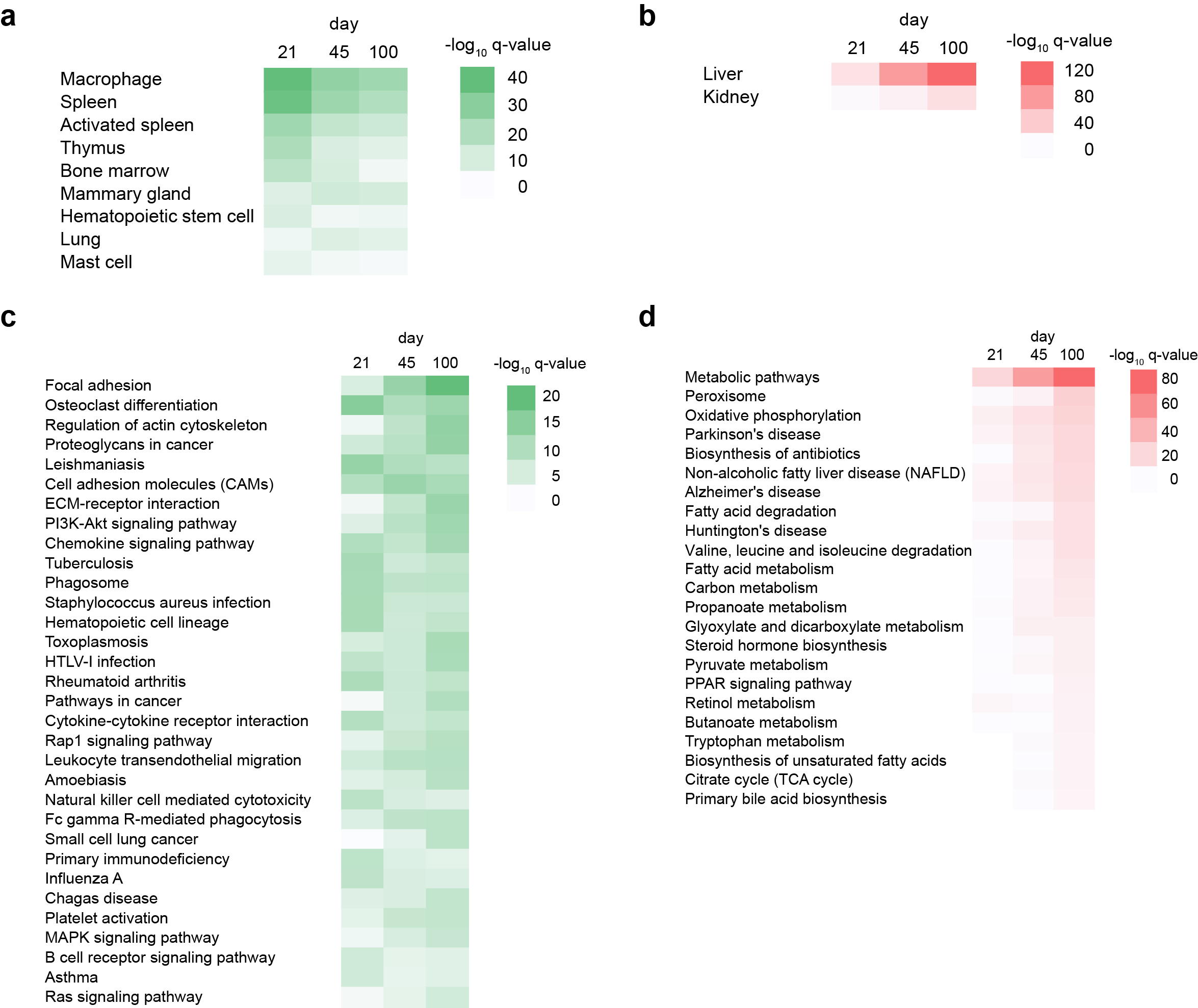


**Supplementary Figure 5** Gene ontology pathway analysis of top pathways impaired at 21, 45 and 100+200 days after Cre removal of miR-122 relative to control mice. **a,b** Tissue expression pattern of genes upregulated (**a**) or downregulated (**b**) in liver samples at various points after miR-122 excision. **c,d** KEGG pathways enriched in genes upregulated (**c**) or downregulated (**d**) after miR-122 excision.

**Supplementary Figure 6** Scenarios for microRNA function in the absence of miR-122.

**
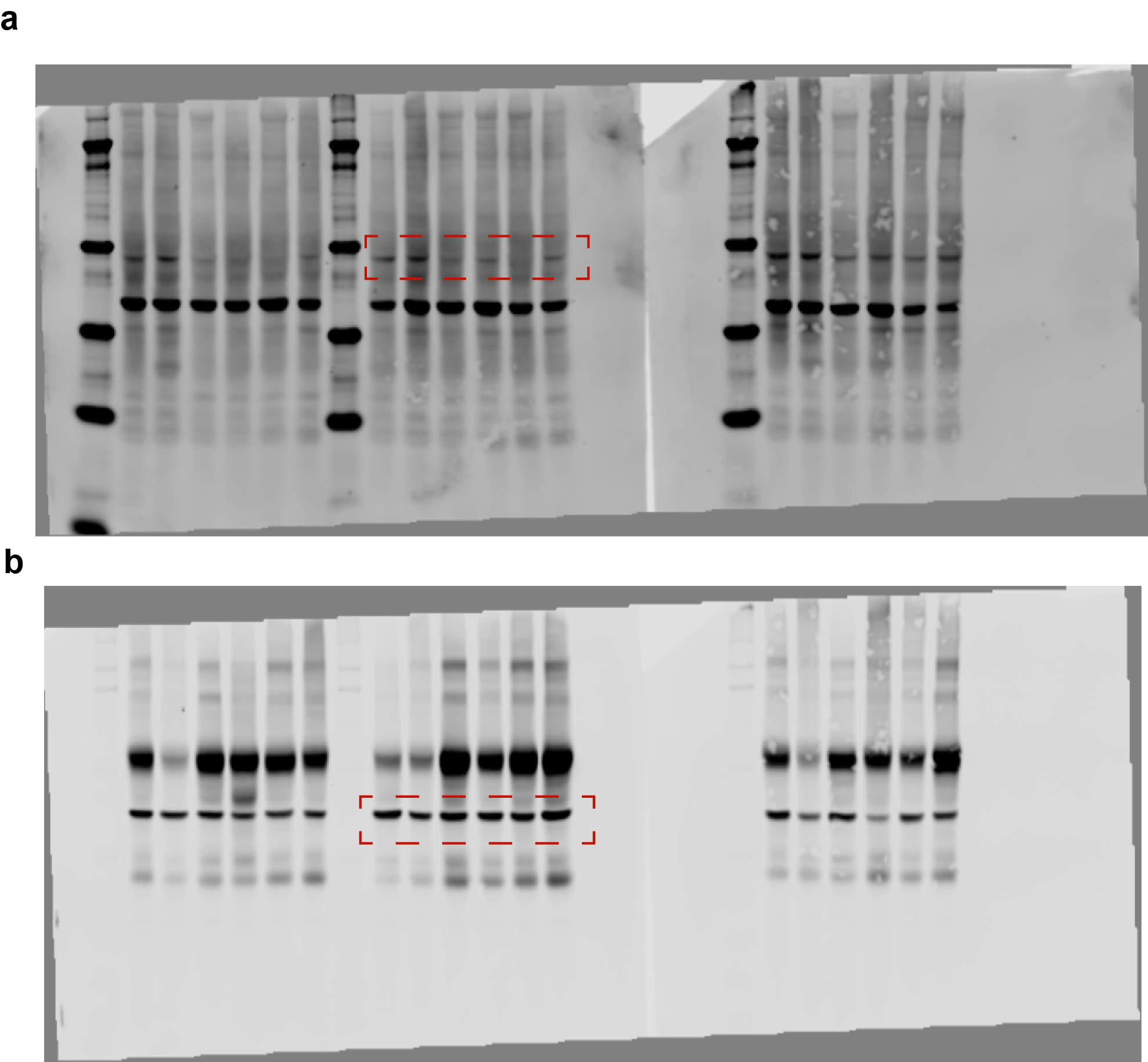
**

**Supplementary Figure 7** Uncropped western blots. Shown are uncropped blots for (**a**) Nr6a1and (**b**) Gapdh.

**Supplementary References**

1. Luna, J.M.*, et al.* Argonaute CLIP Defines a Deregulated miR-122-Bound Transcriptome that Correlates with Patient Survival in Human Liver Cancer. *Mol Cell* **67**, 400-410 e407 (2017).
