# Supplementary material for: miR-122 removal in the liver activates imprinted microRNAs and enables more effective microRNA-mediated gene repression
Source: Nat Commun. 2018 Dec 14;9:5321. doi: 10.1038/s41467-018-07786-7 (PMC6294001; doi:10.1038/s41467-018-07786-7)
Supplement: Supplementary file 3 — Description of Additional Supplementary Files [file 41467_2018_7786_MOESM3_ESM.pdf]

Description of Additional Supplementary Files

File Name: Supplementary Data 1
Description: Normalized microRNA read counts for mouse liver samples

File Name: Supplementary Data 2
Description: RNA sequencing normalized FPKM values for mouse liver samples

File Name: Supplementary Data 3
Description: Differential expression of predicted targets of microRNAs in the mouse liver
